# Incongruity between Affinity Patterns Based on Mandibular and Lower Dental Dimensions following the Transition to Agriculture in the Near East, Anatolia and Europe

**DOI:** 10.1371/journal.pone.0117301

**Published:** 2015-02-04

**Authors:** Ron Pinhasi, Vered Eshed, Noreen von Cramon-Taubadel

**Affiliations:** 1 Earth Institute and School of Archaeology, Belfield, University College Dublin, Dublin, Ireland; 2 Israel Antiquities Authority, Jerusalem, Israel; 3 Department of Anthropology, University at Buffalo, State University of New York, Buffalo, New York, United States of America; University of Oxford, UNITED KINGDOM

## Abstract

While it has been suggested that malocclusion is linked with urbanisation, it remains unclear as to whether its high prevalence began 8,000 years earlier concomitant with the transition to agriculture. Here we investigate the extent to which patterns of affinity (i.e., among-population distances), based on mandibular form and dental dimensions, respectively, match across Epipalaeolithic, Mesolithic, and Neolithic samples from the Near East/Anatolia and Europe. Analyses were conducted using morphological distance matrices reflecting dental and mandibular form for the same 292 individuals across 21 archaeological populations. Thereafter, statistical analyses were undertaken on four sample aggregates defined on the basis of their subsistence strategy, geography, and chronology to test for potential differences in dental and mandibular form across and within groups. Results show a clear separation based on mandibular morphology between European hunter-gatherers, European farmers, and Near Eastern transitional farmers and semi-sedentary hunter-gatherers. In contrast, the dental dimensions show no such pattern and no clear association between the position of samples and their temporal or geographic attributes. Although later farming groups have, on average, smaller teeth and mandibles, shape analyses show that the mandibles of farmers are not simply size-reduced versions of earlier hunter-gatherer mandibles. Instead, it appears that mandibular form underwent a complex series of shape changes commensurate with the transition to agriculture that are not reflected in affinity patterns based on dental dimensions. In the case of hunter-gatherers there is a correlation between inter-individual mandibular and dental distances, suggesting an equilibrium between these two closely associated morphological units. However, in the case of semi-sedentary hunter-gatherers and farming groups, no such correlation was found, suggesting that the incongruity between dental and mandibular form began with the shift towards sedentism and agricultural subsistence practices in the core region of the Near East and Anatolia.

## Introduction

Malocclusion is a condition caused by dental crowding and malposed teeth, that is very common among modern-day world populations, with an average prevalence of 20% [1]. Malocclusion can also be caused by vertical discrepancies, skeletal asymmetries, and other congenital and developmental conditions [2,3]. However, in the great majority of cases it is caused by disparity between jaw size and total tooth-arch length [4]. The condition has been described as the “malady of civilization” [5] and it has been hypothesized that the high prevalence of malocclusion and some of its related conditions (dental crowding, dental rotation) are associated with urbanization and, in some cases, industrialization [6,7]. The main factor believed to underlie this increase in the prevalence of malocclusion is an overall reduction in chewing stress, especially during mandibular and craniofacial growth, resulting in an incongruity between the size of the dental arcade and the jaw [6]. The etiological basis of this incongruity is the capacity of the (osseous) jaw tissue to react during ontogeny to changes in functional demands (phenotypic plasticity) while dental tissue does not remodel in response to biomechanical stress [8].

The relationship between diet, mastication, and occlusal variability in humans is supported by several experimental investigations (cf. [8,9,10], and references therein). It has also been pointed out that the lack of fit between the jaw and dental dimensions cannot be attributed to a reduction in dental wear, for if this were the case then young adults from hunter-gatherer populations with relatively low wear should experience a higher prevalence of malocclusion [7].

The genetic basis of malocclusion is not fully understood due, to some extent, to the lack of clear parameterization of the various morphological components involved. However, estimations of genetic variance and heritability of occlusal variables (i.e. dental, craniofacial, and mandibular dimensions) in monozygotic vs. dizygotic twins shows a considerable amount of environmental influence in the case of most traits [11,12,13], consistent with plastic changes in the upper and lower jaw in response to chewing stress. An array of studies of occlusal variation in various contemporary hunter-gatherer groups that experienced rapid acculturation following contact with western industrial societies indicate that malocclusion is the outcome of non-additive environmental rather than genetic factors in the majority of cases [14,15]. Indeed, increases in the prevalence of malocclusion during the time-span of one generation have been reported for Inuit groups [16,17,18], Native Americans from Florida [19], Australian aborigines [20,21] and South American Indians [22]. Among western societies, a study comparing dental and mandibular dimensions of parents and offspring from 150 English families indicated a secular increase in dental dimensions concurrent with a secular decrease in mandibular dimensions [23]. These opposed secular trends in dental and mandibular size would be consistent with consequent secular increase in the prevalence of malocclusion.

The dentition of hunter-gatherers differs from agriculturalists in several key aspects: larger overall crown size [24,25]; pronounced occlusal and interproximal tooth wear [26,27]; near absence of malocclusion and dental crowing [7,28]; and the low prevalence of certain oral pathologies [8]. Nevertheless, dental crowding was recently reported in an early anatomically modern human from Qafzeh cave, Israel dated to ~100,000 years ago [29], suggesting that malocclusion in humans is not a recent phenomenon. However, the particular period in human prehistory in which dental crowding became a common rather than a rare condition remains to be investigated.

Given the etiological link between chewing (masticatory) stress, diet, and related dental wear patterns [5,30], it seems logical to hypothesise that the time period during which malocclusion became more prevalent lies in the prehistoric transition from hunting/gathering to agriculture. The degree and type of dental wear are affected by the characteristics of the food and by the manner of its preparation [31]. The transition to agriculture is associated with an increased reliance on cooked food, and a reduction in consumption of wild plant and animal resources [24]. The Levant is a region in which agriculture first emerged *in situ* at the onset of the Holocene, ~11,800 cal BP [32]. The transition to agriculture is associated with the Natufian culture (15,000–11,600 cal BP [33]), who were semi-sedentary hunters-cultivators settled within or near woodland. There is palaeobotanical and other archaeological evidence for the intensified cultivation and processing of wild cereals at Natufian sites, as well as investment in permanent structures assumed to be associated with sedentism [34,35]. The most common evidence for intensive processing is the high percentage of sites with stone mortars (70–80% in Natufian sites) [34, 36]. Following the onset of farming during the Pre-Pottery Neolithic A (PPNA, 11,600–10,500 cal BP) there is a shift to a mode of subsistence combining reliance on cultivation of domestic cereals and legumes, the collection of wild seeds and fruits, and hunting [37]. During the subsequent Early, Middle and Late PPNB periods (10,500–8,750 cal BP), the subsistence base shifted to include domestic cereals and a growing reliance on domesticated livestock (cattle, pigs, goats, and sheep). The Pre-Pottery Neolithic C (PPNC, or alternatively Final PPNB, 8,750–8,400 cal BP) was a short transitional period, which is associated with the gradual decline of the PPNB, whether as a response to climatic changes, demographic pressures including diseases, and/or declining yields [33,38].

In a previous study, Pinhasi et al. [39] assessed diachronic reduction patterns in dental and mandibular dimensions of these Levantine populations by analyzing linear regressions of mesiodistal and buccolingal dental dimensions over time. Results indicated a significant reduction in the buccolingual but not mesiodistal dimensions and found changes in ramus breadth and anterior height dimensions of the mandible but not in its overall size. Another study [40] applied the same methods to look at dental reduction patterns among Central European populations from the onset of the Upper Paleolithic until the end of the Neolithic period, showing a different reduction trend in which all slopes of both mesiodistal and buccolingual were significant, but in which the reduction was, on average, 12 times less pronounced than in the buccolingual dimensions of the Levantine populations. These analyses, however, did not compare among-population affinity patterns for the mandibular and dental dimensions and could not detect the particular archaeological period during which these patterns changed, following the transition to agriculture.

Here we investigate between-population distances based on mandibular and dental dimensions in terms of both form (raw data) and shape (size scaled data). Given the vagaries of the available archaeological data for the time period and geographic regions of interest, we also consider statistical patterns of shape and size variation between four broadly defined groups: Upper Palaeolithic and Mesolithic European hunter-gatherers, Neolithic and Eneolithic farmers, and two Levantine transitional groups comprising pre-agricultural (semi-sedentary) hunter-gatherers and transitional farming populations. The assessment of these key chronologically and geographically defined groups allowed us to assess two possibilities. Firstly, was the onset of any incongruity between dental and mandibular form specifically related to the onset of agriculture in the Levant during the Pre-Pottery Neolithic period? Alternatively, is there evidence of incongruity between the dentition and lower jaw across all groups in the Levant and Europe, irrespective of their subsistence strategy? The key benefit of the approach employed here is that the mandibular and dental data are taken from exactly the same individuals. Our *a priori* expectation is that dental and mandibular morphological variation between-groups should reflect the same microevolutionary population history of genetic drift, gene flow, and migration. Therefore, we expect between-group affinity (distance) matrices based on dental and mandibular dimensions to be highly congruent.

## Materials

A dataset of mandibular and crown dimensions was collated from data obtained from a previous study [39] and from previously published sources [41] for 292 Epipalaeolithic, Mesolithic, and Neolithic/Eneolithic samples from the Near East, Anatolia and Europe, spanning a time period between ~28,000–6,000 BP. These samples were assigned to 21 Operational Taxonomic Units (OTUs) based on their broad archaeological, chronological and geographical characteristics (Table 1), such that reasonable sample sizes could be obtained for each OTU.

**Table 1:** Specimens analysed in this study by period, OTU (Operational taxonomic unit) or group.

Location	Country	Lat	Long	N	Arch Period	OTU Name	Age uncal BP	OTU	Group
Dolní Vestonice	Czech	48.88	16.64	4	Early/Mid. UP	E/MUP	28,000–22,000	1	H-G
Grotte des Enfants (Gravettian)	Italy	43.78	7.54	2	Early/Mid. UP	E/MUP	28,000–22,000	1	H-G
Neuessing	Germany	48.9	11.8	1	Early/Mid. UP	E/MUP	28,000–22,000	1	H-G
Predomosti	Czech	49.47	17.44	2	Early/Mid. UP	E/MUP	28,000–22,000	1	H-G
Paglicci	Italy	41.65	15.61	1	Early/Mid. UP	E/MUP	28,000–22,000	1	H-G
Arene Candide (Epi-Gravettian)	Italy	44.17	8.33	5	Late UP	LUP/EMES	13,500–8,500	2	H-G
Bruniquel	France	44.05	1.67	1	Late UP	LUP/EMES	13,500–8,500	2	H-G
Cap Blanc	France	44.93	1.08	1	Late UP	LUP/EMES	13,500–8,500	2	H-G
Farincourt	France	47.70	5.68	1	Late UP	LUP/EMES	13,500–8,500	2	H-G
Le Bichon	Switzerland	47.1	6.87	1	Late UP	LUP/EMES	13,500–8,500	2	H-G
Oberkassel	Germany	50.71	7.16	1	Late UP	LUP/EMES	13,500–8,500	2	H-G
Romito	Italy	39.87	Italy	3	Late UP	LUP/EMES	13,500–8,500	2	H-G
San Teodoro	Italy	38.05	14.58	1	Late UP	LUP/EMES	13,500–8,500	2	H-G
St. Germain La Rivière	France	44.95	-0.33	1	Late UP	LUP/EMES	13,500–8,500	2	H-G
Vado allo Arancio	Italy	43.05	10.89	1	Late UP	LUP/EMES	13,500–8,500	2	H-G
Bonifacio	France	41.39	9.16	1	Mesolithic	LUP/EMES	13,500–8,500	2	H-G
Gough’s Cave	UK	51.28	-2.76	1	Mesolithic	LUP/EMES	13,500–8,500	2	H-G
Kaufertsberg	Germany	48.81	10.61	1	Mesolithic	LUP/EMES	13,500–8,500	2	H-G
Arudy	Spain	43.117	-0.417	1	Mesolithic	LUP/EMES	13,500–8,500	2	H-G
Uzzo	Italy	38.11	12.78	4	Mesolithic	LUP/EMES	13,500–8,500	2	H-G
Molara	Italy	38.08	13.31	2	Mesolithic	LUP/EMES	13,500–8,500	2	H-G
Backäskog	Sweden	56.10	14.35	1	Mesolithic	M/LMES	8,500–6,000	3	H-G
Birsematten-Basisgrotte	Switzerland	47.44	7.55	1	Mesolithic	M/LMES	8,500–6,000	3	H-G
Bottendorf	Germany	51.30	11.40	1	Mesolithic	M/LMES	8,500–6,000	3	H-G
Dragsholm	Denmark	55.77	11.39	1	Mesolithic	M/LMES	8,500–6,000	3	H-G
Koed	Denmark	56.38	10.57	1	Mesolithic	M/LMES	8,500–6,000	3	H-G
Loschbour	Luxemburg	49.75	6.25	1	Mesolithic	M/LMES	8,500–6,000	3	H-G
Montclus	France	43.12	1.24	1	Mesolithic	M/LMES	8,500–6,000	3	H-G
Schellnecker Wand	Germany	48.93	11.83	1	Mesolithic	M/LMES	8,500–6,000	3	H-G
Sejerø	Denmark	55.91	11.09	1	Mesolithic	M/LMES	8,500–6,000	3	H-G
Store Bjers	Sweden	57.80	18.53	1	Mesolithic	M/LMES	8,500–6,000	3	H-G
Vaenge Sø	Denmark	56.13	10.52	1	Mesolithic	M/LMES	8,500–6,000	3	H-G
Skateholm 1	Sweden	55.38	13.48	2	Mesolithic	M/LMES	8,500–6,000	3	H-G
Skateholm 2	Sweden	55.38	13.48	5	Mesolithic	M/LMES	8,500–6,000	3	H-G
Muge Arruda	Portugal	39.11	-8.68	5	Late Mes.	Muge	8,500–6,000	4	H-G
Muge Moita	Portugal	39.11	-8.68	8	Late Mes.	Muge	8,500–6,000	4	H-G
Hoëdic	France	47.35	-2.86	5	Late Mes.	Tev/Hoedic	8,500–6,000	5	H-G
Téviec	France	47.56	-3.16	6	Late Mes.	Tev/Hoedic	8,500–6,000	5	H-G
Eynan	Israel	33.09	35.58	18	Natufian	Eynan	13,000–10,000	6	SSed H-G
Hayonim	Israel	32.90	35.22	9	Natufian	Hayonim	13,000–10,000	7	SSed H-G
Nahal Oren	Israel	32.67	35.00	12	Natufian	Nahal Oren	13,000–10,000	8	SSed H-G
Hohlenstein	Germany	48.55	10.17	2	Mesolithic	Late Mes CE	7,800	9	H-G
Ofnet	Germany	48.82	10.3	12	Mesolithic	Late Mes CE	7,500	9	H-G
Abu Hureyra	Syria	35.87	38.4	3	PPN	PPNA	10,200–9,400	10	Trans F
Hatoula	Israel	31.82	34.98	3	PPNA	PPNA	10,200–9,400	10	Trans F
Çayönü	Turkey	38.23	39.65	2	PPN	PPNA	10,200–9,400	10	Trans F
Jericho	Palestine	31.85	35.45	6	PPN	PPNB	9,500–7,900	11	Trans F
Yiftahel	Israel	32.72	35.18	2	Neolithic	PPNB	9,500–7,900	11	Trans F
Abu Gosh	Israel	31.8	35.12	2	Neolithic	PPNB	9,500–7,900	11	Trans F
Areq el Ahmar	Israel	31.42	35.06	1	PPNB	PPNB	9,500–7,900	11	Trans F
Kefar Hahoresh	Israel	32.7	35.2	4	PPNB	PPNB	9,500–7,900	11	Trans F
Nahal Oren	Israel	32.72	34.97	1	PPNB	PPNB	9,500–7,900	11	Trans F
Atlit Yam	Israel	32.55	34.9	6	PPNC	PPNC	7,900–7,500	12	Trans F
Schwetzingen	Germany	49.38	8.58	14	LBK	Schwetzingen	6,600–6,000	13	Farmers
Viesenhäuser Hof	Germany	48.5	9.13	19	LBK	Vies. Hof	6,600–6,000	14	Farmers
Seehausen	Germany	51.18	11.12	2	LBK	LBK East	6,600–6,000	15	Farmers
Bruchstedt	Germany	51.18	10.78	2	LBK	LBK East	6,600–6,000	15	Farmers
Sonderhausen	Germany	51.38	10.85	4	LBK	LBK East	6,600–6,000	15	Farmers
Radonice-Louny	Czech	49	14	1	LBK	LBK East	6,600–6,000	15	Farmers
Klienfahner	Austria	51.04	10.85	1	LBK	LBK East	6,600–6,000	15	Farmers
Schletz	Austria	48.75	16.47	2	LBK	LBK East	6,600–6,000	15	Farmers
Budakalász	Hungary	47.3	19.03	11	Eneolithic	Baden	4,800–4,200	16	Farmers
Alsónémedi	Hungary	47.18	19.12	2	Eneolithic	Baden	4,800–4,200	16	Farmers
Tjæreby	Denmark	55.24	11.12	2	Funnel Beaker	Dn. M/L Neol	4,600–3,500	17	Farmers
Dragsholm	Denmark	55.48	11.24	1	Funnel Beaker	Dn. M/L Neol	4,600–3,500	17	Farmers
Kærby	Denmark	55.36	11.18	1	Funnel Beaker	Dn. M/L Neol	4,600–3,500	17	Farmers
Lille Havelse	Denmark	55.51	12	1	Funnel Beaker	Dn. M/L Neol	4,600–3,500	17	Farmers
Græse	Denmark	55.54	12.12	1	Funnel Beaker	Dn. M/L Neol	4,600–3,500	17	Farmers
Marbjerg	Denmark	55.42	12.12	3	Funnel Beaker	Dn. M/L Neol	4,600–3,500	17	Farmers
Sønderup	Denmark	55.18	11.18	1	Funnel Beaker	Dn. M/L Neol	4,600–3,500	17	Farmers
Brønhøj	Denmark	56.24	10.48	1	Funnel Beaker	Dn. M/L Neol	4,600–3,500	17	Farmers
Uggerslevgaard	Denmark	55.3	10.18	2	Funnel Beaker	Dn. M/L Neol	4,600–3,500	17	Farmers
Kirke Helsinge	Denmark	55.3	11.18	1	Funnel Beaker	Dn. M/L Neol	4,600–3,500	17	Farmers
Ganløse	Denmark	54.48	12.24	1	Funnel Beaker	Dn. M/L Neol	4,600–3,500	17	Farmers
Rævehøj	Denmark	55.24	11.18	2	Funnel Beaker	Dn. M/L Neol	4,600–3,500	17	Farmers
Udby	Denmark	55	12.18	1	Funnel Beaker	Dn. M/L Neol	4,600–3,500	17	Farmers
Juelsberg	Denmark	55.18	10.48	3	Funnel Beaker	Dn. M/L Neol	4,600–3,500	17	Farmers
Borreby	Denmark	55.12	11.24	5	Funnel Beaker	Dn. M/L Neol	4,600–3,500	17	Farmers
Szegvár-Tüzköves	Hungary	46.29	20.26	1	Tisza Culture	Tisza Culture	6,000	18	Farmers
Kisköre-Gát	Hungary	46.29	20.26	5	Tisza Culture	Tisza Culture	6,000	18	Farmers
Hódmezövásárhely-Kökénydomb	Hungary	46.39	20.39	3	Tisza Culture	Tisza Culture	6,190	18	Farmers
Gumelnita	Romania	44.08	26.63	1	Gumelnita	Gumelnita	5,000–4,400	19	Farmers
Varasti	Romania	44.23	27	13	Gumelnita	Gumelnita	5,000–4,400	19	Farmers
Chamblandes	Switzerland	46.53	6.67	8	Cortalloid	Swiss MN	5,550–5,100	20	Farmers
Collombey Barmaz I	Switzerland	46.27	6.95	3	Middle Neol.	Swiss MN	5,550–5,100	21	Farmers
Collombey Barmaz Ii	Switzerland	46.27	6.95	8	Middle Neol.	Swiss MN	5,550–5,100	21	Farmers
Corseaux	Switzerland	46.28	6.49	5	Middle Neol.	Swiss MN	5,550–5,100	21	Farmers

Abbreviations: uncal BP–uncalibrated years before present; N–total number of individuals included; (L)UP–(Late)Upper Palaeolithic; E/M—Early/Middle; M/L–Middle/Late; MES—Mesolithic; CE- Central Europe; PPN–Pre-Pottery Neolithic; LBK- Linearbandkeramik (Neolithic); MN- Middle Neolithic; Dn.—Danish; Neol.—Neolithic; H-G—Hunter-Gatherers; SSed H-G—Semi-sedentary hunter-gatherers; Trans F—transitional farmers.

Buccolingual and mesiodistal dimensions were recorded in millimetres using the method of Moorrees [42] for the mandibular canines, premolars and molars for each individual. All left-sided teeth were measured except when missing, worn, or poorly preserved. In such cases, the right side antimeres were used instead. Loose dentition that could be assigned to the correct anatomical position was also measured when associated archaeological documentation indicated that it belonged to a single specimen. We excluded all mesiodistal dimensions when inter-proximal wear or pathology resulted in reduction of the overall crown length and buccolingual dimensions when occlusal wear or pathology reduced the overall crown height below the point at which maximum breadth dimension appear to have been located (cf. [43]).

**Fig 1:**
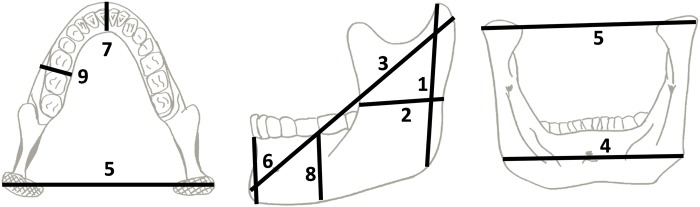
Nine mandibular dimensions recorded following Bräuer (1988). 1. Ramus height (in projection). 2. Ramus breadth. 3. Maximum mandibular length (in projection). 4. Gonial breadth. 5. Bicondylar breadth. 6. Anterior height. 7. Anterior thickness. 8. Corpus height at M1/M2. 9. Corpus thickness at M1/M2.

Nine mandibular dimensions (mm) were measured on each specimen based on the standard methodology specified by Bräuer [44] and based on the system of Martin [45]. See Fig. 1 for a visual description of the measurements taken.

For the purposes of statistical analysis and visualisation of morphological affinity patterns between the mandibular and dental datasets, the 21 OTUs were split into one of four pre-defined groupings (see Table 1). These four groups were: (1) Non-Levantine Upper Palaeolithic and Mesolithic hunter-gatherers (“Hunter-gatherers”). (2) Levantine pre-agricultural semi-sedentary hunter-gatherers (“Semi-sedentary hunter-gatherers”). (3) Levantine Pre-Pottery Neolithic farmers (“Transitional farmers”) and (4) Non-Levantine Neolithic and Eneolithic farmers (“Farmers”).

## Methods

Within each of the two morphological datasets, only those individuals with data present for at least 70% of the measurements were included in the analysis. Importantly, all 292 specimens contributed both mandibular and dental data thereby allowing us to assess any pattern of incongruity between the two datasets. Missing data were estimated for the mandibular and dental datasets separately in SPSS v. 20 using multiple linear regressions, within sexes where possible and using the specimens with a complete set of measurements within each OTU.

The mandibular and dental datasets were separately adjusted for isometric scaling via division of each measurement by the geometric mean of all measurements for that individual specimen [46,47]. Distance matrices for the dental and mandibular datasets were then generated for both the raw (not size-adjusted) data and the new shape variables following the method of Relethford and Blangero [48]. This method uses a quantitative genetic model to estimate pairwise population affinities (distance) under certain assumptions of trait heritability based on the analogous measure developed by Harpending and Ward [49]. Here, we took the conservative approach of assuming complete trait heritability (*h* ^*2*^ = 1), thereby using a minimum estimate of genetic distance among all OTUs. However, it is worth stating that the choice of heritability value does not affect the proportional structure of the resultant distance matrix nor, therefore, the results of any correlations between the matched dental and the mandibular data (see also [50]). Major affinity patterns among OTUs were visualised using principal co-ordinates analysis, with individual OTUs labelled according to the four major groupings described above. The freeware RMET 5.0 was used to construct distance matrices and to conduct subsequent principal co-ordinates analysis.

The strength of correlation between the dental and the mandibular distance matrices was assessed using Mantel tests [51] with 10,000 permutations used to assess significance (α = 0.05) using the software PASSaGE 2.0 (www.passagesoftware.net).

ANOVA with posthoc Bonferroni-adjusted analyses were used to test for differences in the overall size (based on the geometric mean) of the dentition and mandibles among the four pre-defined groups. Given the differences in sample size among the four groups, Levene’s test was first employed to test for significant differences in size variance across groups. Mandibular size (geometric mean) was found not to be significantly different in terms of variance (p = 0.0865) so the parametric ANOVA was employed. Dental size was found to vary significantly among groups (p = 0.276) so the non-parametric Kruskal-Wallis test was employed instead. MANOVA with posthoc Bonferroni-adjusted analyses was used to assess any significant differences in mandibular shape attributes among the four groups. All statistical tests were conducted using SPSS v. 20.

Finally, a post-hoc analysis was performed to assess whether the four pre-defined groups differed in terms of their internal congruence between mandibular and dental form. Between-individual distances were calculated as Euclidean distances based on the mandibular shape data and the raw dental data in PAST v 2.17 [52]. Mantel tests were performed in PASSaGE 2.0 to assess the correlation between the mandibular and dental inter-individual distance matrices.

## Results

**Fig 2:**
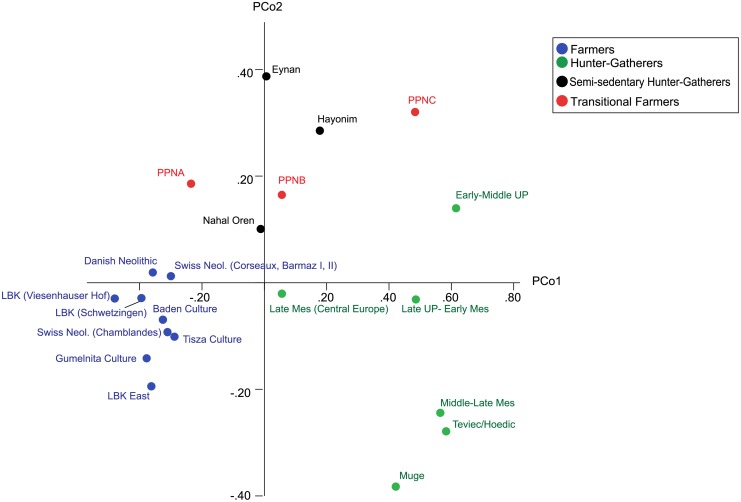
Plot of the first two principal co-ordinates (explaining 62.6 and 16.9% of variation, respectively) based on mandibular form (raw data).

The plot of the first two principal coordinates for mandibular form (raw data) indicates a separation based on mandibular morphology between all hunter-gatherers (positive PCO1 scores) and European agriculturalists (negative PCO1 scores) (Fig. 2). PCO2 separates between the Levantine transitional farmers and pre-agricultural hunter-gatherers and the European hunter-gatherers and farmers. The plot of the first two principal coordinates for the size-adjusted mandibular shape variables shows the same basic pattern (S1 Fig.).

**Fig 3:**
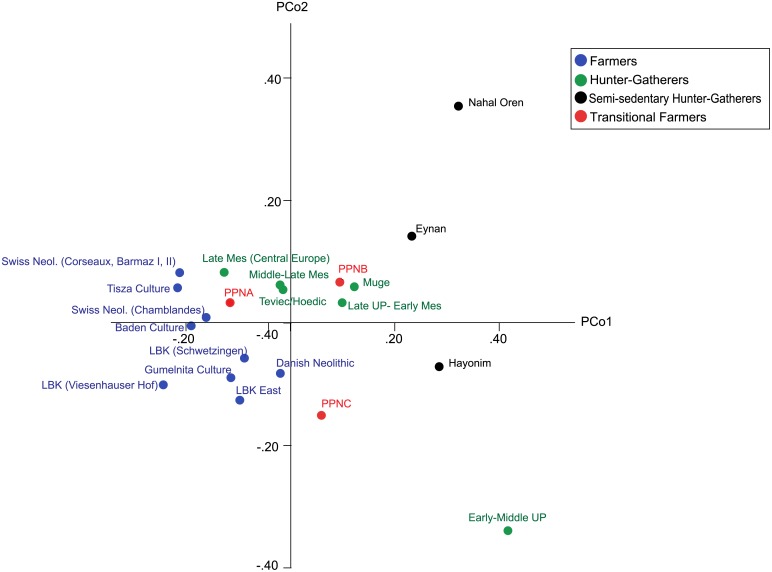
Plot of the first two principal co-ordinates (explaining 46.7 and 23.7% of variation, respectively) based on raw mesiodistal and buccolingual dental dimensions.

Principal co-ordinates analysis of the raw dental dimensions did not show the same pattern seen in the case of the mandibular dimensions (Fig. 3). There is a general decline in overall size (PCO1) with smaller dental dimensions among European farmers and larger dimensions among the remaining three dietary and geographic groups. This result is supported by the results of a Kruskal-Wallis test which showed that tooth size is significantly different among the four main groups, with posthoc Mann Whitney U analyses (Bonferroni adjusted) finding that all three pre-farming and transitional groups had significantly larger dentition than the farmers, but were not significantly different from each other in terms of dental size variation (Table 2). The results of the ANOVA carried out on mandibular size was also significant (p < 0.001) with post-hoc analyses showing that the two hunter-gatherer groups and the transitional farmers all had significantly larger mandibles than the farmers, although there was no significant differences among the three pre- or transitional farming groups (Table 2).

**Table 2:** Results of Kruskal-Wallis test of dental size and the ANOVA of mandibular size differences.

Dental		H-G	SSed H-G	TransFarmers	Farmers
Size		*8.65*	*8.79*	*8.57*	*8.31*
	H-G				
	SSed H-G	0.525			
	TransFarmers	1.000	0.115		
	Farmers	**<0.001**	**<0.001**	**0.002**	

Mandible		H-G	SSed H-G	TransFarmers	Farmers
Size		*41.97*	*42.38*	*43.79*	*40.20*
	H-G				
	SSed H-G	1.000			
	TransFarmers	0.674	1.000		
	Farmers	**<0.001**	**<0.001**	**<0.001**	

In both cases the initial cross-group comparison was highly significant (p<0.001). Here Bonferroni- adjusted posthoc analyses show the pairwise differences among the four groups. Dental and mandibular size (in italics) was quantified using the geometric mean of all dimensions in that dataset. Significantly different comparisons (α = 0.05) are in bold.

**Fig 4:**
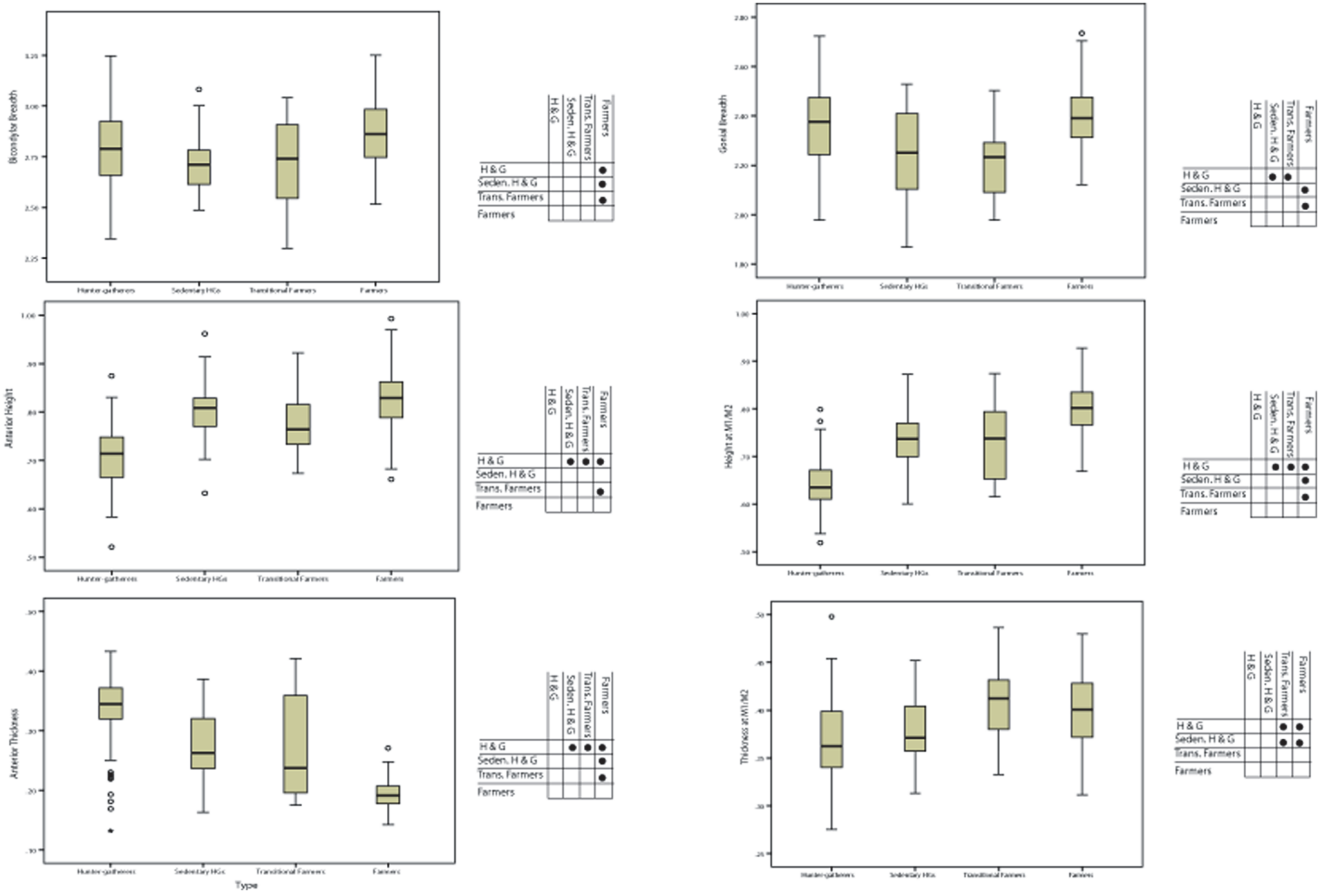
Composite of box plots for six of the mandibular shape variables among the four pre-defined subsistence and chronological groups. In each case the pairwise dot table shows which groups were significantly different according to the posthoc results of the MANOVA (Table 3).

The results of the Mantel tests found no significant relationship between population distances based on dental form and mandibular form (r = 0.224, p = 0.218) nor between population distances based on dental shape and mandibular shape (r = 0.0631, p = 0.541). As might be expected, the only distance matrices that were significantly correlated were dental shape and form (r = 0.854, p = 0.0001) and mandibular shape and form (r = 0.991, p = 0.0001). The results of the MANOVA were also significant showing shape differences among the four groups (Pillai’s Trace p<0.001; Wilk’s Lambda p<0.001). Post-hoc tests applying Bonferroni correction show a complex pattern of affinity based on mandibular shape (Table 3). Fig. 4 illustrates the major shape differences among the four groups. It is clear from these results that although farmers have (on average) smaller mandibles than the hunter-gatherer and transitional farming OTUs this is not an instance of isometric scaling as the patterns of shape transformations differ depending on which variable is being considered. The only two variables where farmers differ significantly to the exclusion of all other groups are Ramus Height and Bicondylar Breadth. Farmers have relatively taller rami and relatively broader mandibles at the condyles (Table 3). In some cases (Fig. 4) the trend is a relative decrease/increase in the size of certain variables in the two Levantine groups (e.g. Gonial Breadth) with the European hunter-gatherers resembling the European farmers most closely. However, in a few variables there is a clear trend in terms of average differences among groups from hunter-gatherer through to fully farming populations. In particular, Anterior Height is significantly larger among farmers and semi-sedentary hunter-gatherers than among hunter-gatherers and transitional farmers. Corpus Height at M1/M2 shows a similar pattern with a clear increase in relative height for the farmers when compared to all three non-farming groups. In contrast, Anterior Thickness of the mandibular body is significantly reduced over time, but this is not the case for Corpus Thickness at M1/M2 which is smaller for both hunter-gatherer groups in comparison to the transitional farmers and farmers. In fact, this latter variable (Corpus Thickness at M1/M2) is the only variable that is not significantly different between farmers and transitional farmers, and distinguishes the two agricultural groups from the two purely hunter-gatherer groups.

**Table 3:** Results of MANOVA comparisons of mandibular shape variables among the four groups.

1.Ramus height		H-G	SSed H-G	TransFarmers	Farmers
		*1.38*	*1.38*	*1.39*	*1.46*
	H-G				
	SSed H-G	1.000			
	TransFarmers	1.000	0.168		
	Farmers	**<0.001**	**0.001**	**0.006**	

2.Ramus breadth		H-G	SSed H-G	TransFarmers	Farmers
		*0.80*	*0.85*	*0.83*	*0.79*
	H-G				
	SSed H-G	**<0.001**			
	TransFarmers	0.176	0.746		
	Farmers	0.414	**<0.001**	**0.003**	

3.Mandibular length		H-G	SSed H-G	TransFarmers	Farmers
		*2.53*	*2.37*	*2.42*	*2.56*
	H-G				
	SSed H-G	**<0.001**			
	TransFarmers	**0.002**	1.000		
	Farmers	1.000	**<0.001**	**<0.001**	

4.Gonial breadth		H-G	SSed H-G	TransFarmers	Farmers
		*2.36*	*2.25*	*2.20*	*2.40*
	H-G				
	SSed H-G	**0.001**			
	TransFarmers	**<0.001**	1.000		
	Farmers	0.406	**<0.001**	**<0.001**	

5.Bicondylar breadth		H-G	SSed H-G	TransFarmers	Farmers
		*2.80*	*2.71*	*2.72*	*2.87*
	H-G				
	SSed H-G	0.060			
	TransFarmers	0.170	1.000		
	Farmers	**0.021**	**<0.001**	**<0.001**	

6.Anterior height		H-G	SSed H-G	TransFarmers	Farmers
		*0.71*	*0.80*	*0.77*	*0.83*
	H-G				
	SSed H-G	**<0.001**			
	TransFarmers	**<0.001**	0.401		
	Farmers	**<0.001**	0.112	**<0.001**	

7.Anterior thickness		H-G	SSed H-G	TransFarmers	Farmers
		*0.34*	*0.27*	*0.28*	*0.19*
	H-G				
	SSed H-G	**<0.001**			
	TransFarmers	**<0.001**	1.000		
	Farmers	**<0.001**	**<0.001**	**<0.001**	

8. Height at M1/M2		H-G	SSed H-G	TransFarmers	Farmers
		*0.64*	*0.74*	*0.73*	*0.80*
	H-G				
	SSed H-G	**<0.001**			
	TransFarmers	**<0.001**	1.000		
	Farmers	**<0.001**	**<0.001**	**<0.001**	

9.Thickness at M1/M2		H-G	SSed H-G	TransFarmers	Farmers
		*0.37*	*0.38*	*0.41*	*0.40*
	H-G				
	SSed H-G	1.000			
	TransFarmers	**<0.001**	**0.008**		
	Farmers	**<0.001**	**0.032**	1.000	

In the case of all nine mandibular variables the initial MANOVA was significant (p<0.001). Here Bonferroni- adjusted posthoc analyses show the pairwise differences among the four groups for each of the nine variables. Average (size-adjusted) values for each of the four groups are given in italics for each variable. Significantly different comparisons (α = 0.05) are in bold.

The results of the post-hoc analysis of inter-individual congruence in mandibular shape and dental form found that hunter-gatherers demonstrated a significant correlation (r = 0.150, p = 0.013), while all the other three groups did not; semi-sedentary hunter-gatherers (r = -0.056, p = 0.520), transitional farmers (r = 0.149, p = 0.071) and farmers (r = 0.053, p = 0.232).

## Discussion

No correlation was found between OTU affinity (distance) patterns based on dental and mandibular dimensions, showing a fundamental mismatch between these two morphological units, despite the fact that the data were taken from the same individual specimens. However, the results of the post-hoc analysis suggest that this mismatch may be attributable to the onset of semi-sedentism and farming practices in the Near East. In hunter-gatherers there is a correlation between among-individual differences in mandibular shape and dental form, suggesting an “equilibrium” in the association between the lower jaw and lower dentition. This is logical and consistent given the fundamentally close morphological and functional association between these two anatomical units. Our results suggest that the lack of correlation between dentition and mandibular distance matrices begins with the shift towards sedentism and proceeds in a mosaic fashion throughout the transition to agriculture in the Levant. This is in accord with previous results indicating a difference in the magnitude and type of crown reduction trend in the Near East/Anatolia [39] and Europe [40]. While farmers have (on average) smaller teeth and mandibles than all non-farming groups, farmers’ mandibles are not simply size-scaled versions of the larger mandibles of chronologically older groups; they are scaled allometrically. As evinced by the results of the MANOVA analyses, some mandibular dimensions are getting relatively larger while others are getting relatively smaller, indicative of a mosaic pattern of mandibular shape change throughout the transition to agriculture.

Although the system of shape quantification is substantially different from the 3D geometric morphometric protocols used by von Cramon-Taubadel [53], some overlap in results can be identified. von Cramon-Taubadel [53] employed a 3D analysis of mandibular shape to show that contemporary farming and hunter-gatherer populations differ in their average mandibular morphology in a manner consistent with differences in their subsistence strategies. Farmers were found, on average, to have relatively taller rami and coronoid processes, relatively shorter mandibles anteriorly-posteriorly and relatively wider mandibles across the corpus and ramus [53]. Our results are consistent with these findings in so far as the farmers were found to have significantly taller rami and significantly wider mandibles at the condyles. Unfortunately, it was not possible to assess differences in relative mandibular length as the variable employed here (maximum mandibular length in projection) is measured in such a way that it includes the relative length of the ramus, the relative breadth of the ramus, and the relative length of the corpus. Also, it is worth noting that it was not possible to specifically quantify the relative length of the dental arch using this dataset, which prevented the direct assessment of mismatch between tooth size and space for dental eruption in the case of the present study. Hence, further 3D shape analysis combined with integrated dental crown dimension data would be required to disentangle the possible effects of the transition to agriculture on each individual aspect of mandible shape.

What is clear from these analyses, however, is that mandibular shape was changing throughout the transition to agriculture, and different aspects of mandibular form were changing at different points in time. Our results hint that such a mosaic morphological pattern occurred with changes in settlement patterns and associated subsistence practices, which is important for understanding the potential impact of the transition to agriculture on the relative form of the mandible and the relative size of the dentition. Dental size, along with overall body size [54], decreases across the transition to agriculture with farming populations having overall smaller teeth and smaller body size than earlier hunter-gatherer populations. Yet, the mandibular form of farmers is not isometrically smaller relative to earlier hunter-gatherers. Some dimensions are relatively larger (e.g. ramus height), while others may be decreasing as part of a general trend in size reduction starting as early as the Natufian (semi-sedentary hunter-gatherers) period. This is consistent with the notion that the mandible is a highly plastic bone, capable of substantial shape change throughout ontogeny in direct response to biomechanical stress [8,10,53].

The notion that malocclusion is absent or very rare in pre-industrial populations has its roots in both the anthropological and dental literature based on the study of modern subjects or historical (mainly medieval) populations [29]. However, some studies report reduction in occlusal abnormalities and overall wear pattern when comparing Archaic hunter-gatherers with Mississippian agriculturalists from Koger’s Island in the Pickwick Basin, Tennessee [55] and when assessing diachronic patterns among Greek populations from the Neolithic to modern times [56]. The comparison of a 16th century graveyard population with modern orthodontic data for Scandinavians showed an increase in the prevalence of malocclusion from 36% in the medieval population to 65% in the modern population. Similar prevalence rates were also reported for other studies (e.g. [57]).

Another consideration in the assessment of potential malocclusion in prehistoric populations is the level of dietary variation and in particular the amount of chewing of hard foods [58]. A temporal assessment of 15 archaeological skeletal series from Japan [59] comprising skulls of pre-agricultural Early (8,000–5,000 BP), and Late (4000–2200 BP) Jomon Period; early farming Yayoi Period (2200–1800 BP) and protohistoric Kofun Period (1800–1400 BP) show an increase in the prevalence of malocclusion from 22.2% in Early Jomon and 20.5% in Late Jomon, to 48.8% in Yayoi and 36% in Kofun [59]. These findings do not suggest any temporal trend in Japan, but do show a major increase in the prevalence of malocclusion among the agricultural populations which Inoue et al. [59] have attributed to the use of soft foods requiring less chewing force following the transition to farming in Japan.

The degree of occlusal attrition is directly related to the coarseness of the diet, dietary consistency, mandibular developmental plasticity, and the amount of interstitial wear caused by enamel rubbing on enamel as the teeth move up and down in their sockets during mastication [4,60,61,62,63]. It therefore follows that inter-population variation in diet will result in corresponding variation in the prevalence of malocclusion. Variation in diet and related mastication will result in corresponding variation in dental wear. Angled molar wear patterns are typical of agriculturalists while flat molar wear is characteristic of hunter-gatherers [64]. In the southern Levant, a comparison of dental wear patterns among three populations–Natufian hunter-gatherers (10,500–8300 BC) from Ain Mallaha, pre-Pottery Neolithic B (PPNB) population from Kefar HaHoresh and PPNC population from Atlit Yam–show an extensive amount of inter-site differences in wear patterns and oral pathology [65] which corresponds to variations in food preparation techniques, amount of sand in the diet, and differences in the consumption of terrestrial versus marine resources. This variability is reflected in our mandibular form analysis (Fig. 2) indicating a high degree of morphological variability among the three pre-Pottery Neolithic populations (transitional farmers), which fall out alongside the Natufian populations (semi-sedentary hunter-gatherers) and intermediate between European hunter-gatherer and farming populations.

## Conclusions

Our study investigated time-specific patterns in the incongruity between dental (crown) dimensions and mandibular dimensions among groups spanning the Upper Palaeolithic to the Bronze Age. The analyses showed a separation based on mandibular morphology between hunter-gatherers, farmers and Near Eastern semi-sedentary hunter-gatherers and transitional farmers (Fig. 2). Results of the same analysis carried out on dental dimensions show no such pattern and no clear association between the position of populations in multidimensional space and their temporal or geographic attributes (Fig. 3). Moreover, no significant correlations were found between population distances based on mandibular morphology and dental dimensions, despite representing exactly the same individual skeletons. Within hunter-gatherers, there was a correlation between inter-individual differences in mandibular shape and dental form, but no such correlation was found amongst the other three groups. These results suggest a different pattern of inter-population variation in dental and mandibular dimensions, hinting at the fact that the lack of correlation between dental size and mandibular form that is associated with various types of malocclusion among modern-day populations, began to arise with the shift towards sedentism and agricultural subsistence practices several millennia ago.

## Supporting Information

S1 Fig Plot of the first two principal co-ordinates (explaining 62.1 and 14.9% of variation, respectively) based on mandibular shape. (EPS)

S1 Table A complete set of all the mandibular and dental dimensions analysed in this study. (XLS)
